# Sagittal Position of Nail in Talus Affects Tibiotalocalcaneal Arthrodesis Outcomes

**DOI:** 10.1177/19386400251346518

**Published:** 2025-06-24

**Authors:** Sean O’Leary, Navneet K. Venugopal, Ariadna Robledo, Roshan B. Tom, Daniel C. Jupiter, Jie Chen, Vinod K. Panchbhavi

**Affiliations:** John Sealy School of Medicine, The University of Texas Medical Branch, Galveston, Texas; John Sealy School of Medicine, The University of Texas Medical Branch, Galveston, Texas; Department of Internal Medicine, University of Miami, Miami, Florida; John Sealy School of Medicine, The University of Texas Medical Branch, Galveston, Texas; Department of Orthopaedic Surgery, The University of Texas Medical Branch, Galveston, Texas; Department of Biostatistics & Data Science, The University of Texas Medical Branch, Galveston, Texas; Department of Orthopaedic Surgery, The University of Texas Medical Branch, Galveston, Texas; Department of Orthopaedic Surgery, The University of Texas Medical Branch, Galveston, Texas

**Keywords:** arthrodesis, hindfoot, surgical implants, talus, tibiotalocalcaneal nails

## Abstract

**Background:**

Tibiotalocalcaneal (TTC) nails are commonly used implants for hindfoot arthrodesis. This study evaluated the relationship between the sagittal placement of the nail—specifically, more anterior versus more posterior positioning within the talus—and patient outcomes following surgery.

**Methods:**

We conducted a retrospective, single-center study involving patients aged 18 years and older who underwent hindfoot nail fusion. Variables analyzed included smoking status, body mass index (BMI), diabetes mellitus (DM) status, age, cause of surgery, surgical complications including nonunion, and need for revision surgery. Postoperative X-rays were assessed to calculate the talar-to-nail ratio (tnR), defined as the distance between the anterior margin of the talus and the center of the hindfoot nail (nL) relative to the total length of the talus (tL), expressed as tnR = nL/tL.

**Results:**

The study included 53 patients (24 male, 29 female) aged 18-92 years. The mean tnR was 54.635% (SD = 9.925), with a range from 23.89% to 76.71%. Bivariate analysis showed no significant relationship between the analyzed variables and nonunion or revision surgery, with the exception of tnR, which was significantly positively associated with revision surgery (59.130 ± 8.253 vs 51.912 ± 9.967, P = .009). In multivariable logistic regression, while DM and age were not significantly associated with revision surgery (P > .05), tnR demonstrated a significant association, with an odds ratio of 1.093 (95% CI: 1.017-1.193) and a P-value of .026.

**Conclusion:**

More posterior nail placement, as indicated by a higher tnR, is significantly associated with an increased likelihood of revision surgery, potentially due to an increased foot lever arm. These findings suggest that more anterior nail placement may lead to improved surgical outcomes in TTC arthrodesis.

**Level of Evidence:**

Level III, Prognostic

“More posterior nail placement, reflected by a higher talar-to-nail ratio, was significantly associated with increased revision surgery rates following hindfoot arthrodesis.”

## Introduction

Tibiotalocalcaneal (TTC) arthrodesis results in the complete immobilization of the ankle and hindfoot, which significantly limits overall foot function.^1-3^ Despite this limitation, TTC arthrodesis is a valuable surgical technique for alleviating pain and enhancing stability by creating a stable, plantigrade foot, which is essential for ambulation.^4,5^ The procedure involves retrograde insertion of a nail through the calcaneus, secured with interlocking screws. This approach, utilizing small incisions, minimizes soft tissue damage and lowers the risk of infection.^6^ Consequently, TTC arthrodesis is often reserved as a last-resort treatment for severe dysfunction and arthritis affecting both the ankle and subtalar joints, as well as for managing persistent deformities associated with conditions such as rheumatoid arthritis, severe posttraumatic arthritis, Charcot arthropathy, talar avascular necrosis, or paralysis.^7^

Patients undergoing this “last resort” procedure frequently present with decreased bone mineral density and multiple comorbidities, making precise nail placement critical for reducing stress risers and achieving adequate fixation.^8^ Recent studies have demonstrated significant improvements in mobility (76.9%) and a high satisfaction rate of nearly 95% following hindfoot arthrodesis, emphasizing its effectiveness in this challenging patient population.^9,10^

Despite its benefits, TTC arthrodesis is associated with nonunion rates of 20% or higher, underscoring the difficulties in achieving stable fixation and successful bony union.^11-14^ In this study, we investigated the relationship between nail placement, focusing on more anterior versus more posterior positioning relative to the talus, and patient outcomes, specifically revision surgery. This analysis aimed to identify optimal surgical techniques that may enhance the success of TTC arthrodesis.

## Methods

### Study Design and Population

This retrospective cohort study was conducted at a single-center hospital and included patients aged 18 years and older who underwent hindfoot nail fusion between 2012 and 2024. The study included patients with postoperative radiographs and at least 1 follow-up visit. Patients were excluded if they had incomplete radiographic data, insufficient follow-up (defined as less than 1-week postoperatively), or underwent additional procedures unrelated to the hindfoot fusion. This study was reviewed by our institution’s Institutional Review Board and was deemed exempt from review (IRB # 23-0281).

### Data Collection

Demographic and clinical data were extracted from the hospital’s electronic medical records system. Collected variables included patient age at index surgery, sex, body mass index (BMI), smoking status (categorized as current, former, or never smoker), and comorbidities, such as diabetes mellitus (DM), hypertension, chronic kidney disease, end-stage renal disease, and peripheral artery disease. Surgical details, including the side of arthrodesis (left or right) and the primary indication for the procedure (e.g., fracture, degenerative joint disease, and Charcot foot), were documented.

### Radiographic Analysis

Postoperative radiographs were analyzed using the Picture Archiving and Communication System (PACS). The primary radiographic measurement of interest was the talar-to-nail ratio (tnR), which quantifies the sagittal position of the nail within the talus. The tnR was calculated by measuring the distance between the anterior margin of the talus and the center of the hindfoot nail (nL), then normalizing this distance by the total length of the talus (tL), resulting in the ratio tnR = nL/tL. To ensure accuracy, the tnR was measured by 2 independent observers and discrepancies were resolved by consensus.

### Outcome Measures

The primary outcomes assessed included postoperative complications such as nonunion, revision surgery, wound infection, and other adverse events directly involving the surgical site, including necrosis, numbness, and swelling, which could occur at any time following surgery. In addition, the occurrence of revision surgeries and the time to revision surgery were recorded.

### Statistical Analysis

Descriptive statistics were used to summarize patient demographics, comorbidities, and postoperative outcomes. Continuous variables were reported as mean values with standard deviations (SDs) and ranges, while categorical variables were expressed as frequencies and percentages.

To explore associations between tnR and postoperative outcomes, bivariate analyses were conducted using independent *t* tests or chi-square tests, depending on whether the variables were continuous or categorical. Statistical significance was defined as *P* < .05. A multivariable logistic regression analysis was conducted to adjust for potential confounders, specifically targeting variables with a bivariate *P*-value of less than .1. The primary goal of the regression analysis was to evaluate the association between tnR and the likelihood of requiring revision surgery and likelihood of nonunion. Results from the logistic regression were presented as odds ratios (ORs) with 95% confidence intervals (CIs) and corresponding *P*-values.

### Operative Technique

Patients were positioned in the lateral decubitus position with a proximal thigh tourniquet applied to achieve hemostasis. Following sterile preparation and draping, a lateral incision was utilized over the distal fibula. A fibular osteotomy was made to access the tibiotalar and subtalar joints. All remaining cartilage and interposed scar tissue was removed with osteotomes, curettes, and rongeurs. Following this, the joints were fenestrated with a 2.0-mm drill and the joint was provisionally pinned in neutral position at the ankle and hindfoot. Next, a plantar incision was made, and blunt dissection was used to access the calcaneus. A guidewire was advanced across the subtalar and ankle joints into the distal tibial plafond, followed by sequential reaming. A 200-mm × 10-mm intramedullary nail was then inserted, and interlocking screws were placed proximally and distally. The surgical sites were irrigated and closed in layers.

Postoperatively, patients were kept nonweightbearing for 6 weeks in a splint, followed by an additional 4 to 6 weeks in a fracture boot. Follow-up visits were scheduled at 3, 6, and 12 weeks, with additional evaluations at 3- to 6-month intervals as needed. Anteroposterior and lateral weightbearing radiographs were obtained at each visit, with computed tomography scans ordered only if nonunion was suspected. Radiographic union was defined by full callous formation or cortical bridging across the ankle and subtalar joints, with no lucency between the fusion surfaces. Figure 1 presents representative radiographs from 2 included patients, illustrating an anteriorly placed nail with a tnR of 23.89% (Figure 1A), followed by the postoperative outcome (Figure 1B), and a posteriorly placed nail with a tnR of 76.71% (Figure 1C), with the corresponding postoperative outcome (Figure 1D).

**Figure 1.**
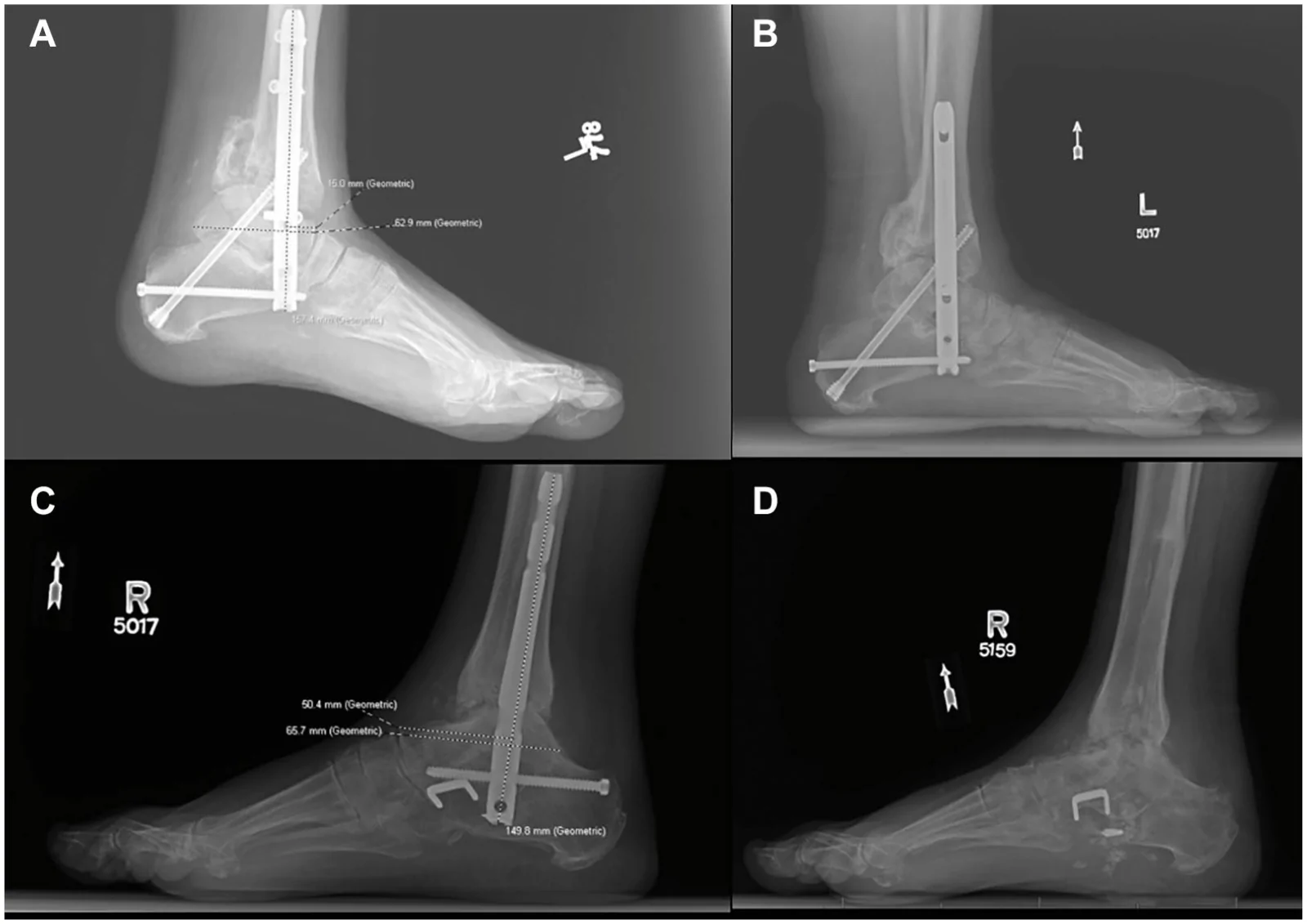
(A) Anteriorly placed nail in a patient, with nL = 15 mm, tL = 62.9 mm, and tnR = 23.89%. (B) Follow-up imaging demonstrates successful healing and union. (C) Posteriorly placed nail in another patient, with nL = 50.4 mm, tL = 65.7 mm, and tnR = 76.71%. (D) Follow-up imaging shows hardware removal with near-complete joint space loss, bone resorption, and cystic changes.

## Results

### Clinical Characteristics

The demographic and clinical characteristics of the 53 patients included in the final analysis are summarized in Table 1. The mean age of the cohort was 53.42 ± 14.16 years, with a range of 18 to 92 years. The cohort consisted of 24 men (45.3%) and 29 women (54.7%). Arthrodesis was performed on the left side in 19 patients (35.8%) and on the right side in 34 patients (64.2%). The mean BMI was 32.4 ± 6.9. The most common comorbidities were hypertension (n = 29, 54.7%) and DM (n = 21, 39.6%). Thirteen patients (24.5%) were current smokers, and an equal number were former smokers. All patients were followed up for a mean time of 488.6 ± 433.4 days, range 27 to 1544 days.

**Table 1.** Clinical Characteristics, Medical History, Indications for Tibiotalocalcaneal Arthrodesis, Radiographic Measurements, and Postoperative Outcomes for the 53 Patients Included in the Study.

	Variable	Count (%)/Mean ± SD
Clinical characteristics	Patients	53
	Male	24 (45.3%)
	Female	29 (54.7%)
	Age	53.4 ± 14.2
	Left	19 (35.8%)
	Right	34 (64.2%)
History	Current smoker	13
	Former smoker	13
	Body mass index	32.4 ± 6.9
	Hypertension	29 (54.7%)
	Deep vein thrombosis	3 (5.7%)
	Diabetes mellitus	21 (39.6%)
	Chronic kidney disease	8 (15.1%)
	End-stage renal disease	2 (3.8%)
	Peripheral artery disease	3 (5.7%)
Surgical history	Previous hindfoot surgery	35 (66%)
Indications	Fractures	20 (37.7%)
	Degenerative joint disease	15 (28.3%)
	Charcot foot	8 (15.0%)
	Hindfoot valgus	2 (3.8%)
	Equinus contracture	2 (3.8%)
	Pes cavus	1 (1.9%)
	Flail ankle	2 (3.8%)
	Cavovarus deformity	1 (1.9%)
	Calcaneal malalignment	1 (1.9%)
Radiographic	Talar-to-nail ratio	54.6% ± 9.9%
Outcome	Pain	45 (84.9%)
	Numbness	3 (5.7%)
	Necrosis	1 (1.9%)
	Infection	12 (22.6%)
	Wound drainage	12 (22.6%)
	Nonunion	13 (24.5%)
	Wound dehiscence	5 (9.4%)
	Operative site swelling	17 (32.1%)
	Follow-up (days)	488.6 ± 433.4
Reoperation	Revision surgery	20 (37.7%)
	Time to follow-up surgery (days)	615.2 ± 509.3

The indications for TTC arthrodesis included fractures being the most frequent indication (n = 20, 37.7%), followed by degenerative joint disease (n = 15, 28.3%) and Charcot foot (n = 8, 15%). A history of previous lower extremity surgery on the affected foot was reported in 35 patients (66%) (Table 1).

### tnR and Postoperative Outcomes

Radiographic measurements and postoperative outcomes are reported in Table 1. The mean tnR was 54.64% ± 9.93% (range, 23.89%-76.71%). The mean time to the first postoperative follow-up was 1.8 ± 0.8 weeks. Postoperatively, the most commonly reported issue was pain (n = 45, 84.9%), followed by infection (n = 12, 22.6%) and wound drainage (n = 12, 22.6%). Follow-up surgery was required in 26 patients (49%), with a mean time to revision surgery of 615.2 ± 509.3 days (range, 60-1544 days).

### Bivariate and Multivariate Analysis

The results of bivariate analysis of DM, sex, and smoking status on nonunion and the need for revision surgery are reported in Table 2. The analysis revealed no statistically significant associations between these variables and nonunion or the need for revision surgery, as indicated by *P*-values greater than .05 in all comparisons.

**Table 2.** Bivariate Analysis of Categorical Variables (Diabetes Mellitus, Sex, and Smoking Status) in Relation to Postoperative Outcomes for the 53 Patients Included in the Study.

Variable	Nonunion (count)	No nonunion (count)	*P*	Revision surgery (count)	No revision surgery (count)	*P*
Diabetes mellitus						
Yes	3	18	.239	5	16	.090
No	9	23		15	17	
Sex						
Male	7	17	.302	8	16	.547
Female	5	24		12	17	
Smoking status						
Current	5	8	.246	6	7	.734
Former	3	10		5	8	
Never	4	23		9	18	

Denotes statistical significance (*P* < .05).

The results of bivariate analysis of the continuous variables age, BMI, and tnR on nonunion and the need for revision surgery are summarized in Table 3. While age and BMI were not significantly associated with nonunion or the need for revision surgery (*P* > .05), a statistically significant difference was observed for tnR in relation to revision surgery (*P* = .02). This finding suggests that tnR may be a relevant factor in determining the likelihood of requiring revision surgery.

**Table 3.** Bivariate Analysis of Continuous Variables (Age, Body Mass Index, and Talar-to-Nail Ratio) in Relation to Postoperative Outcomes for the 53 Patients Included in the Study.

Variable	Nonunion (mean ± SD)	No nonunion (mean ± SD)	*P*	Revision surgery (mean ± SD)	No revision surgery (mean ± SD)	*P*
Age (years)	47.25 ± 12.505	55.22 ± 14.24	.091	52.55 ± 15.042	53.939 ± 13.807	.905
Body mass index	34.427 ± 4.338	31.750 ± 7.433	.170	32.364 ± 7.394	32.350 ± 6.719	.783
Talar-to-nail ratio	56.522 ± 5.421	54.083 ± 10.890	.406	59.130 ± 8.253	51.912 ± 9.967	.02*

Abbreviations: SD = standard deviation.

* Denotes statistical significance (*P* < .05).

Table 4 presents the findings from the multivariate analysis examining the relationships between DM and tnR with the likelihood of revision surgery, as well as age and nonunion. The multivariate logistic regression model indicated that age was not significantly associated with nonunion (*P* = .093). Similarly, in the multivariable logistic regression model adjusted for both DM and tnR, DM was not significantly associated with the likelihood of revision surgery (*P* = .240). However, tnR was significantly associated with revision surgery (OR 1.093, 95% CI 1.017-1.193, *P* = .026).

**Table 4.** Multivariate Analysis of Diabetes Mellitus and Talar-to-Nail Ratio Regarding the Need for Revision Surgery and Age Regarding Nonunion in the 53 Patients Included in the Study.

Variable	Odds ratio	CI (lower bound—upper bound)	*P*
Revision surgery			
Diabetes mellitus	0.239	0.113-1.658	.240
Talar-to-nail ratio	0.406	1.017-1.193	.026*
Nonunion			
Age	0.958	0.906-1.005	.093

Abbreviations: CI = confidence interval.

* Denotes statistical significance (*P* < .05).

## Discussion

The surgical management of hindfoot and ankle instability, particularly through TTC arthrodesis, poses significant challenges due to the high complication rates often associated with the procedure.^11-14^ In this study, we explored the relationship between the tnR and postoperative complications, with a particular focus on the need for revision surgery. Our findings demonstrated a statistically significant association between a higher tnR and an increased likelihood of revision surgery, emphasizing the importance of the sagittal positioning of the nail within the talus as a critical determinant of surgical success.

We hypothesize that the relationship between a lower tnR and a reduced need for revision surgery is due to the decreased lever arm associated with more anterior nail placement. The tnR was calculated to provide a standardized metric that accounts for variations in the dimensions of the calcaneus and talus relative to the tibia (Figure 2A). Mathematically, the lever arm is expressed as lever arm = *r*sinθ, where *r* represents the distance (or radius) from the axis of rotation (in this case, the joint between the talus and tibia that forms the ankle joint) to the point where force is applied.^15^ A lower tnR corresponds to a shorter radius, and consequently, a shorter lever arm (Figure 2B). With a reduced lever arm, less force is transmitted to the ankle joint as torque, which can be represented by the equation torque = force × lever arm.^15^ This reduction in torque lowers the mechanical stress on the implanted hardware, decreasing the likelihood of complications such as hardware loosening or failure, which often necessitate revision surgery and revision arthrodesis. In contrast, a more posterior nail placement (resulting in a higher tnR) would increase the lever arm, thereby amplifying the torque and potentially increasing the risk of nonunion or hardware-related complications as seen in our results.

**Figure 2.**
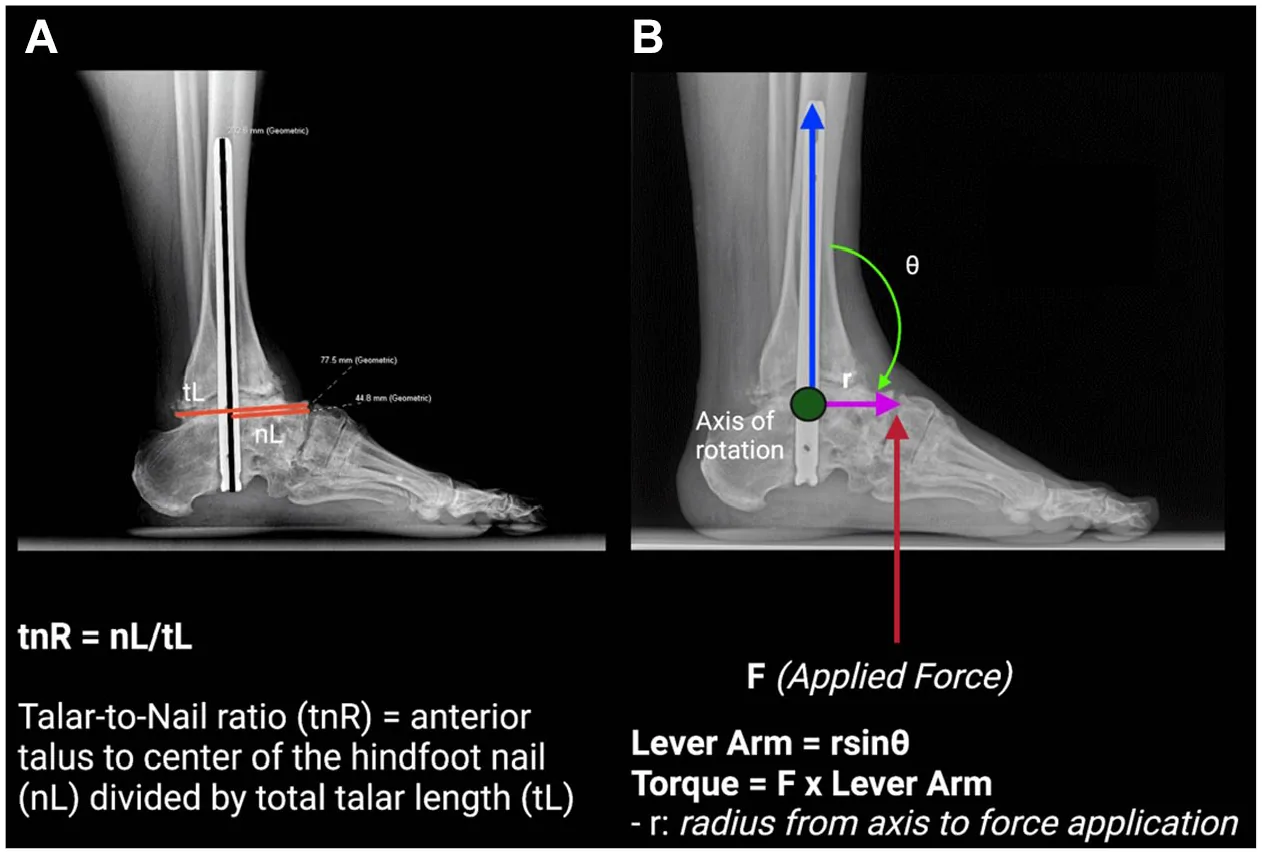
(A) Radiograph illustrating the measurement of the talar-to-nail ratio (tnR), with total talar length (tL) and the distance from the anterior talus to the center of the hindfoot nail (nL) indicated by horizontal red lines. The talar nail is marked by a vertical black line. (B) Diagram showing the lever arm and torque forces applied to the ankle joint.

Currently, there is a lack of quantitative research on optimal sagittal nail placement in TTC arthrodesis. Burgesson et al^16^ discussed the traditional preference for posterior nail placement in cavus foot deformities to enhance calcaneal compression, with medial placement often chosen for its alignment with the tibial mechanical axis and ease of insertion. However, our study suggests achieving an optimal tnR is crucial for reducing hardware-related complications and improving overall patient outcomes in TTC arthrodesis.

In a related work, Dominic Marley et al^17^ explored the impact of nail length and curvature on the central positioning within the tibia and reported that improper positioning can lead to increased cortical stress and subsequent complications such as cortical hypertrophy and stress fractures. Their findings indicated that angles of incidence and reflection were greater with short, straight nails (e.g., 15 cm), reducing as nail length increased and approaching zero with long curved nails (e.g., 20 cm). Specifically, cortical hypertrophy was observed in 54% of patients with short, straight nails, compared with only 7% with long, curved nails, and stress fractures were exclusively reported in patients with straight nails.^17^ Their study supports the use of curved nails to achieve better overall outcomes in TTC arthrodesis. Building on this, Fang et al^18^ demonstrated that curved nails not only maintain physiological valgus but also reduce neurovascular risk, resulting in a 100% union rate with an average time to union of 3.9 months and minimal complications, including only 1 case of delayed wound healing and no reported cases of stress fractures or significant cortical hypertrophy. Our findings extend this understanding by demonstrating that not only coronal, but also sagittal positioning of the nail, particularly a more anterior placement, can significantly reduce the risk of revision surgery due to decreased mechanical stress.

Previous research has indicated that patients with diabetes have poorer outcomes in hindfoot fusion procedures, likely due to impaired bone healing and increased susceptibility to infection.^19^ Similarly, although smoking has been identified as a risk factor for postoperative complications, with studies showing up to a 2.7 times higher relative risk of complications in smokers, our study did not find a significant correlation.^20^ Within our specific study population, these associations were not found with diabetes or smoking status, strengthening the conclusion that tnR is predictive of outcome in the absence of other potential confounding factors.

Our study has several limitations, including a discrepancy in follow-up duration between the entire cohort (488.6 ± 433.4 days) and the subgroup undergoing revision surgery (615.2 ± 509.3 days). This discrepancy is likely due to patients who experienced complications requiring revision surgery, necessitating longer follow-up periods compared with those without the need for revision. Prospective clinical trials are necessary to validate these findings and refine current clinical guidelines. Future research should focus on investigating the biomechanical effects of nail positioning, particularly the influence of tnR on joint stability and fusion success. Additionally, incorporating curved nails into sagittal analyses may further optimize tnR and potentially enhance outcomes in TTC arthrodesis.

## Conclusion

The findings of this study underscore the critical influence of the tnR on the outcomes of TTC arthrodesis. Our results indicate that a higher tnR is significantly associated with an increased risk of revision surgery, highlighting the importance of meticulous nail positioning within the talus. These insights suggest that careful attention to tnR during surgical planning and execution is essential for minimizing postoperative complications and optimizing patient outcomes. Given these findings, we recommend incorporating tnR considerations into TTC arthrodesis surgical guidelines to enhance procedural success.

## Data Availability

Not applicable.
